# Genetic signature detected in T cell receptors from patients with severe COVID-19

**DOI:** 10.1016/j.isci.2023.107735

**Published:** 2023-08-25

**Authors:** Manuel Corpas, Carmen de Mendoza, Víctor Moreno-Torres, Ilduara Pintos, Pedro Seoane, James R. Perkins, Juan A.G. Ranea, Segun Fatumo, Tamas Korcsmaros, José Manuel Martín-Villa, Pablo Barreiro, Octavio Corral, Vicente Soriano

**Affiliations:** 1 School of Life Sciences, University of Westminster, London, UK; 2 Cambridge Precision Medicine Limited, ideaSpace, University of Cambridge Biomedical Innovation Hub, Cambridge, UK; 3 UNIR Health Sciences School & Medical Center, Madrid, Spain; 4 Institute of Continuing Education, University of Cambridge, Cambridge, UK; 5 Puerta de Hierro University Hospital & Research Institute, Majadahonda, Spain; 6 Department of Molecular Biology and Biochemistry, University of Málaga, Málaga, Spain; 7 CIBER de Enfermedades Raras (CIBERER), Instituto de Salud Carlos III, Madrid, Spain; 8 The Biomedical Research Institute of Málaga (IBIMA), Málaga, Spain; 9 Spanish National Bioinformatics Institute (INB/ELIXIR-ES), Madrid, Spain; 10 The African Computational Genomics (TACG) Research Group, MRC/UVRI and LSHTM, Entebbe, Uganda; 11 London School of Hygiene and Tropical Medicine, London, UK; 12 H3Africa Bioinformatics Network (H3ABioNet) Node, Centre for Genomics Research and Innovation, NABDA/FMST, Abuja, Nigeria; 13 Faculty of Medicine, Department of Metabolism, Digestion and Reproduction, Imperial College London, London, UK; 14 Department of Immunology, Complutense University and IIS Gregorio Marañón, Madrid, Spain; 15 Emergency Hospital Isabel Zendal, Madrid, Spain

**Keywords:** Genetics, Immunology, Virology, Genomics

## Abstract

Characterization of host genetic factors contributing to COVID-19 severity promises advances on drug discovery to fight the disease. Most genetic analyses to date have identified genome-wide significant associations involving loss-of-function variants for immune response pathways. Despite accumulating evidence supporting a role for T cells in COVID-19 severity, no definitive genetic markers have been found to support an involvement of T cell responses. We analyzed 205 whole exomes from both a well-characterized cohort of hospitalized severe COVID-19 patients and controls. Significantly enriched high impact alleles were found for 25 variants within the T cell receptor beta (TRB) locus on chromosome 7. Although most of these alleles were found in heterozygosis, at least three or more in *TRBV6-5*, *TRBV7-3*, *TRBV7-6*, *TRBV7-7*, and *TRBV10-1* suggested a possible TRB loss of function via compound heterozygosis. This loss-of-function in TRB genes supports suboptimal or dysfunctional T cell responses as a major contributor to severe COVID-19 pathogenesis.

## Highlights

• T cell receptor inactivated genes appear enriched in severe COVID-19 patients
• These genes participate in highly specific antigen recognition
• Results suggest suboptimal T cell responses in severe SARS-CoV-2 infection

## Graphical abstract

**Figure:**
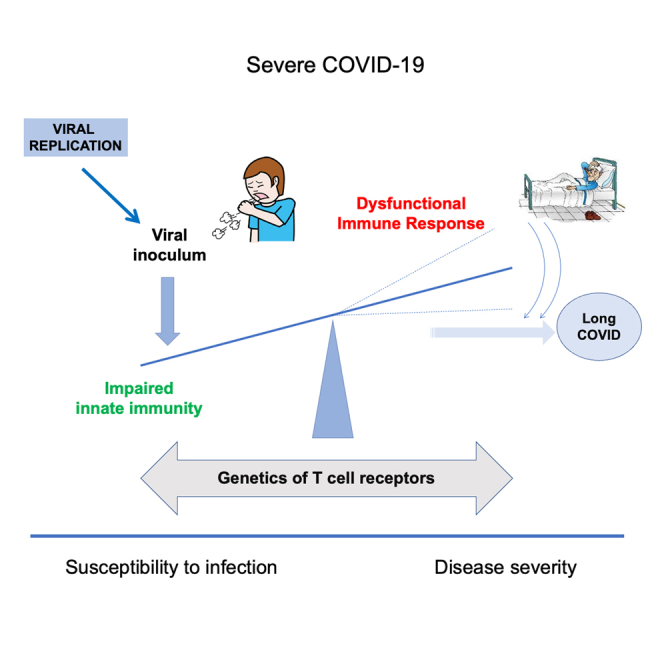

## Introduction

During March 2020, Spain experienced a rapid surge of COVID-19 cases, making Madrid one of the epicenters of Europe’s first pandemic wave. Within two months, a large temporary patient outflow hospital was set up at the city’s international convention center, with hundreds of beds lined up in pavilions normally used as auditoriums and exhibition halls. Many of the patients taken there were in critical condition and a high proportion unfortunately died.^1^ Although acute respiratory disease syndrome (ARDS) was a common clinical feature, other clinical complications, including thromboembolic events, were identified as contributors to COVID-19 disease severity.^2^

Many studies have tried to characterize the determinants of clinically severe COVID-19. However, it is not well understood why some patients become critically ill while others hardly show any symptoms. Some host factors such as older age, male sex, and comorbidities (e.g., diabetes, obesity, cancer, or clotting disorders) have shown to significantly increase the chances of developing severe COVID-19.^3^^,^^4^

The international COVID-19 Host Genetics Initiative^5^^,^^6^ and others^7^^,^^8^^,^^9^^,^^10^^,^^11^^,^^12^^,^^13^^,^^14^ have identified several loci associated with enhanced susceptibility to SARS-CoV-2 infection and/or disease severity.^15^ These studies have provided a greater understanding of the mechanisms for COVID-19 disease pathogenesis. However, as more fine-grained phenotypic descriptions become available, together with deeper genetic sequencing from ancestrally diverse patients, new genetic associations are expected to arise. Such associations may provide key insights for better recognition and prioritization of the most vulnerable patients, enabling the application of precision medicine approaches.^16^ Individualized strategies for patient prioritization may include a broader range of interventions, including earlier prescription of oral antivirals,^17^^,^^18^ repeated vaccine boosters, social isolation measures, etc.

A wide number of genetic variants have been associated with severe COVID-19, frequently pointing at immune response dysfunction.^19^^,^^20^ Among the most relevant genetic factors for immune dysfunction are genes located in the major histocompatibility complex (MHC). The human leukocyte antigen (HLA), MHC in humans, consists of a group of genes located on the short arm of chromosome 6. They encode surface glycoproteins of two types, HLA class I or II molecules, with different tissue distribution and molecular characteristics. This system is extremely polymorphic, with multiple genes and allelic variants, although a given individual possesses only two alleles inherited in a Mendelian fashion. The main function of the HLA molecules is to distinguish foreign invaders such as viruses and bacteria from the body’s own cells. Pathogen-derived peptides (anchored in the HLA molecule) are presented to T lymphocytes which, in turn, are activated, exerting their immune function. T lymphocytes engage with HLA through a surface receptor (T cell receptor, TCR). These receptors are generated in the thymus by a random rearrangement mechanism. Here genes from a group of TCRA and TCRB segments (in the case of αβTCR) or TCRG and TCRD segments (in the case of γδTCR) stochastically mobilize segments to generate TCR receptors. These rearrangements facilitate the development of a large repertoire of diverse cells, enabling them to protect against distinct infections. Prior to exerting their function in the periphery, these newly arranged TCR must be assayed against the HLA molecules present in the thymus. Only those able to establish cognate interactions with the molecules will survive and exit to the periphery. Given the polymorphism of the HLA system and the stochastic rearrangement of TCR segments, the final TCR repertoire available to confront pathogens differs between individuals.

TCR gene alleles have long been considered immune response genes, but evidence has been lacking for diseases involving complex antigens like whole microorganisms and broad tissue autoantigens. Obvious relationships have been found at the level of individual pathogen peptides or autoantigen peptides. The HLA alleles are the prototypical immune response genes. However, they rarely impact on immune responses against pathogens, although exceptions exist like HIV. The HLA protective alleles HLA-B∗27, HLA-B∗57, and HLA-B∗58:01 present immunodominant peptides such as Gag protein-restricted by HLA-B∗27. TCR is a disulfide-linked membrane-anchored complex consisting of the highly variable alpha and beta chains bound to the invariant CD3 chain. The variable domain of TCR alpha-chain and beta-chain have three complementarity-determining regions. The complementarity-determining region of the beta-chain is encoded in locus q34 of chromosome 7 and has been shown to interact with antigens with a high degree of specificity.^21^ The role of HLA and TCR on COVID-19, however, remains unclear.

Herein, we report a genetic study performed on highly selected patients with severe COVID-19 hospitalized during the first wave in Madrid, Spain, before the introduction of vaccines. Our patient cohort was compiled following strict clinical inclusion criteria, including age younger than 60 years, no comorbidities, and hypoxemic bilateral pneumonia. Controls were ancestry matched and bioinformatically processed in an identical manner to avoid batch effect biases. Our study yielded 25 high impact variants (21 frameshifts and 4 stop codons) at genome-wide significance (p value >5.0E-8) within the TRB locus of the q34 band in chromosome 7. Genes *TRBV6-5*, *TRBV7-3*, *TRBV7-6*, *TRBV7-7*, and *TRBV10-1* contained at least 3 high impact alleles in heterozygosis from different variants, suggesting a possible mechanism of TRB loss of function via compound heterozygosis. Our results support a role of T cell receptors via loss of function in the exacerbation of COVID-19 symptoms, potentially leading to suboptimal and/or dysfunctional immune responses to SARS-CoV-2 infection as a major determinant of disease severity.

## Results

### Description of clinical phenotypes

**Figure 1:**
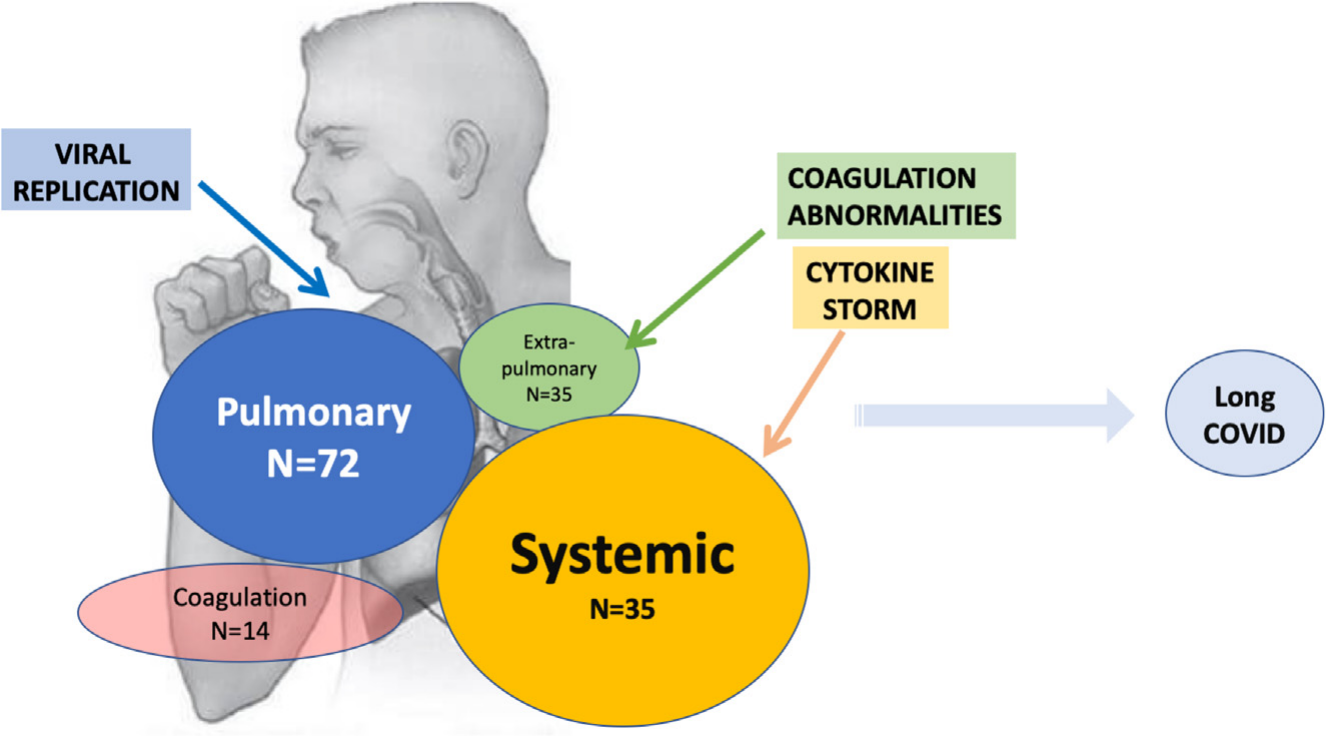
Number of patients affected with symptoms in our highly selected population Determinants of severe COVID-19 and major clinical phenotypes with numbers referring to patients with severe COVID-19 in our study population within each group.

Figure 1 shows the four major clinical phenotypes of severe COVID-19 and the number of patients that exhibited conditions within each group. Briefly, from the 74 cases, pulmonary manifestations were recorded in 72, extra-pulmonary conditions in 35, coagulation disorders in 14, and systemic manifestations in 35.

Table 1 describes the set of 28 clinical terms split into the 4 overarching COVID-19 major clinical phenotypes along with the specific number of patients affected (right column). By decreasing order, the phenotypes affecting more than 10 patients were pneumonia (N = 72), acute respiratory syndrome disease (ARDS; N = 42), persistent fever (N = 30), ARDS and intensive care unit (ICU; N = 19), fatigue, malaise, headache and arthromyalgia (N = 13 each), and hepatitis (N = 11). None of our patients developed stroke, peripheral arterial thrombosis, arthritis, seizures, or myelitis.

**Table 1:** List of medical terms for COVID-19 clinical manifestations

Phenotype	Phenotype ID	Patient Number (N = 74)
Pneumonia	1.	72
ARDS	2.	42
ARDS & ICU	3.	19
Skin—exanthema	4.	5
Heart—myocarditis	5.	1
Heart—arrhythmia	6.	3
Liver—hepatitis	7.	11
Kidney—glomerulonephritis	8.	0
Kidney—tubulopathy	9.	4
Neurological—encephalitis/encephalopathy	10.	7
Neurological—psychiatric (delirium, etc.)	11.	7
Neurological—polyneuropathy (neuropathy, Guillain-Barré, etc.)	12.	7
Neurological—myelitis	13.	0
Neurological—seizure	14.	0
Gastrointestinal—diarrhea	15.	8
Gastrointestinal—nausea/vomiting	16.	4
Endocrine dysfunction (thyroid, etc.)	17.	0
Musculoskeletal—myopathy	18.	1
Musculoskeletal—arthritis	19.	0
Bone marrow—blood cytopenia, pancytopenia/aplasia	20.	6
Pulmonary embolism	21.	8
Deep venous thrombosis	22.	4
Peripheral arterial thrombosis	23.	0
Stroke	24.	0
Ischemic heart event	25.	1
Disseminated intravascular coagulation	26.	3
Persistent fever	27.	30
Fatigue, malaise, headache, arthromyalgia	28.	13

A total of 28 terms were defined across 4 broad categories of symptoms: Pulmonary, Extrapulmonary, Coagulation, and Systemic. Each of our patients with severe COVID-19 was assessed for each term. The right column provides a count of the number of patients affected within a selected set of 74 cases.

Patients with severe COVID-19 were additionally sorted by the number of major COVID-19 phenotypes, which somewhat acted as a proxy for a greater number of symptoms. Interestingly, the top 19 patients with the greatest number of COVID phenotypes were all males.

### Analysis of high impact variants

**Figure 2:**
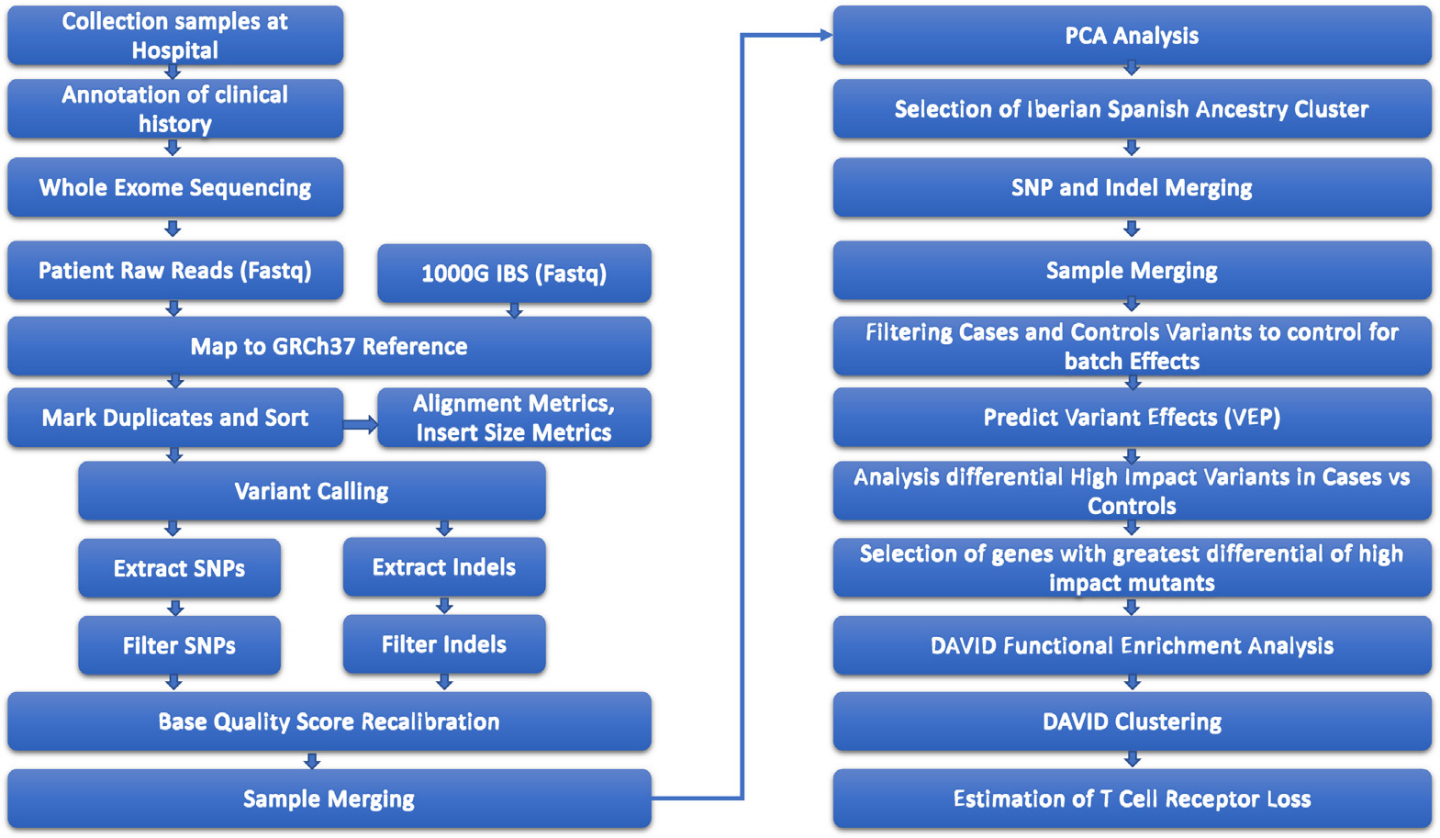
Flow chart illustrating samples origin, filtering, and data analysis (Bioinformatics workflow) Here, we describe the different steps taken to analyze the patient data and come up with our variants of concern.

**Figure 3:**
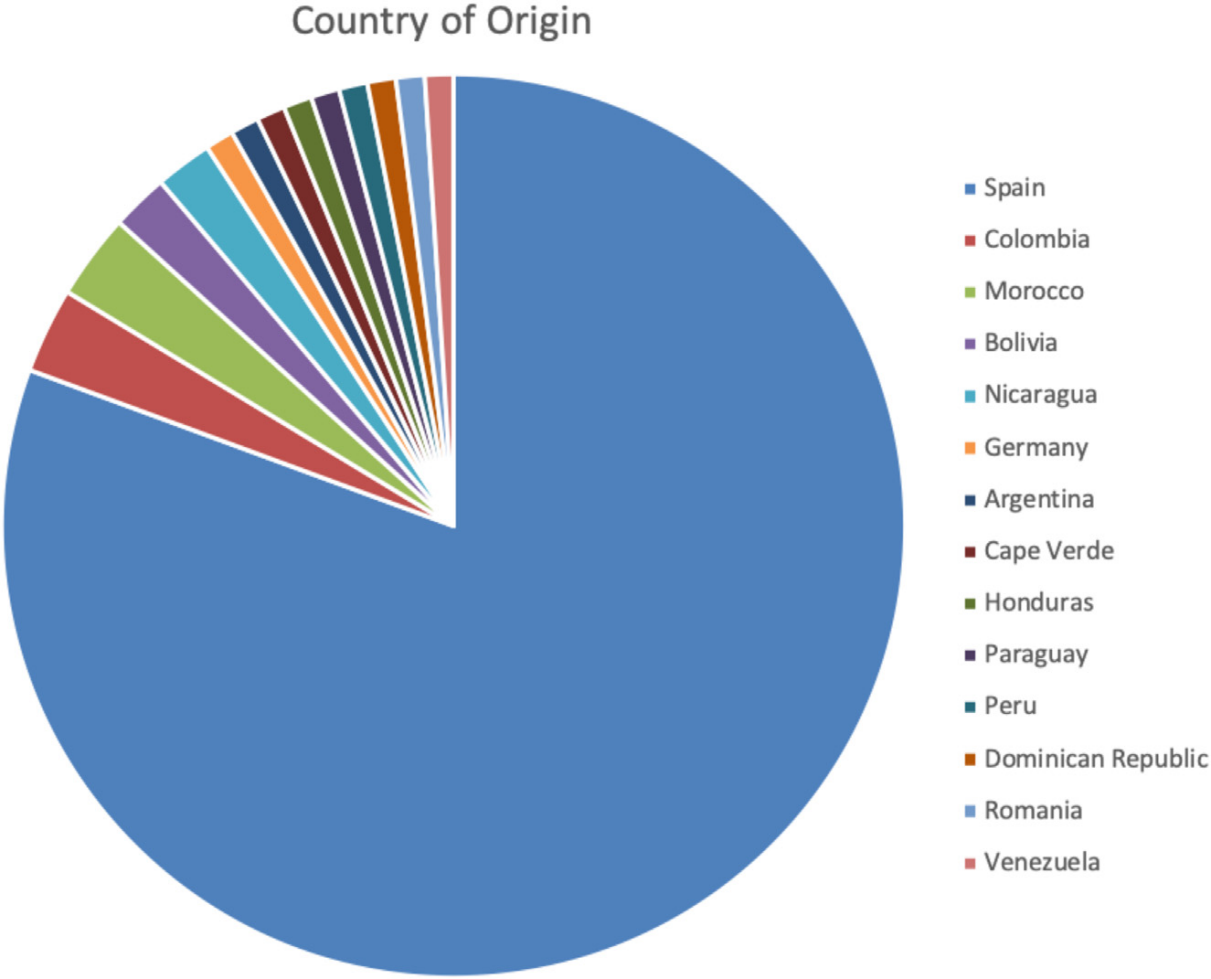
Country of birth for the 98 cases with severe COVID-19 enrolled in the study (Case Cohort) Overall 19 individuals were born outside of Spain.

Figure 2 illustrates samples origin, filtering, and data analysis. Figure 3 describes the initial break down of country of origin from samples before filtering those that did not cluster within our controls' Iberian Spanish (IBS) genetic distance. A total of 851,386 variants were identified in the joint cohort of 167 severely affected cases (N = 74) and controls (N = 93). Overall 32,366 (3.80%) were novel variants. The total number of high impact variants was 5,589, averaging 322 per exome. Of note, 1,477 high impact variants were rare (i.e., not present in gnomAD^22^), averaging 53 per exome. Table 2 summarizes these numbers.

**Table 2:** Summary statistics of all genetic variants analyzed in the study population

	Cases and controls (N = 167)
Variants processed	851,386
Average variants processed/exome	134,706
Novel variant occurrences	32,366
Novel variants (%)	3.80
Existing/known variants	819,020
Existing variants (%)	96.20
High impact	5,589
Exome average high impact	322
Rare high impact	1,477
Exome average rare high impact	53

From a total of 851,386 common variants in cases and controls 5,589 were of high impact, an average of 322 per exome.

### Case-control high impact differentially affected genes

We identified high impact variants predicted by Variant Effect Predictor (VEP).^23^ Genes containing high impact variants were further selected for analysis as long as both case and control samples were affected in the same gene (to avoid case-control batch effects due to different coverages). This yielded a total of 1,119 genes containing at least 1 high impact mutation in both cases and controls. We identified significantly different case/control genes with high impact mutations (Bonferroni-corrected p value = 0.05/1,118 = 4.47E-05). Table 3 records genes with p values below this significance threshold. Overall, 12 out of the resulting 60 genes (20%) are T cell receptor genes. We carried over this list of 60 genes for functional enrichment analysis.

**Table 3:** Number of cases and controls with high impact variants within a gene (as identified by Ensembl’s Variant Effect Predictor)

Gene Name	Cases (N = 74)	Controls (N = 93)	P Value
*ADCK5*	52	2	3.29E-13
*AKR1C3*	67	8	2.63E-13
*ALDH3B2*	2	27	6.55E-07
*ANKDD1B*	58	6	3.12E-12
*ANKRD36*	70	17	8.24E-10
*B3GNT6*	70	31	1.73E-05
*C4orf50*	31	1	1.39E-08
*CASP12*	70	4	2.10E-16
*CCDC30*	22	2	1.20E-05
*CLDN5*	57	22	1.57E-05
*CNTNAP3*	22	2	1.20E-05
*CNTNAP3B*	53	18	5.95E-06
*COL6A5*	66	4	2.08E-15
*EPB41L4A*	22	1	2.77E-06
*FAM157A*	30	2	1.14E-07
*FAM182B*	53	6	5.18E-11
*FOXD4L3*	52	2	3.29E-13
*GALNT9*	33	1	4.30E-09
*GOLGA6L2*	70	31	1.73E-05
*HERC2*	61	14	4.65E-09
*IGHV3-64*	28	1	8.07E-08
*LENG9*	63	5	4.79E-14
*LTN1*	36	8	5.24E-06
*MAL2*	70	7	1.36E-14
*MUC5B*	32	2	3.58E-08
*NOTCH2*	31	3	2.61E-07
*NPIPB15*	65	18	2.28E-08
*OPLAH*	70	11	1.81E-12
*OR10D3*	57	10	6.45E-10
*OR11H7*	47	4	1.09E-10
*OR4C5*	70	30	9.98E-06
*OR5G3*	53	15	5.87E-07
*PLK5*	31	2	6.40E-08
*PRAMEF2*	55	12	1.45E-08
*SIX1*	69	3	8.38E-17
*SLC9B1*	68	28	6.94E-06
*TRAJ37*	70	19	4.78E-09
*TRAV19*	30	5	5.48E-06
*TRBV10-1*	70	29	5.64E-06
*TRBV30*	40	8	6.45E-07
*TRBV5-5*	70	23	1.12E-07
*TRBV6-5*	70	21	2.45E-08
*TRBV6-7*	53	13	1.03E-07
*TRBV7-1*	43	10	9.84E-07
*TRBV7-3*	70	22	5.30E-08
*TRBV7-6*	70	12	5.53E-12
*TRBV7-7*	70	3	7.22E-19
*TRBV7-8*	34	1	2.40E-09
*UBXN11*	69	7	2.39E-14
*UNKL*	57	21	8.21E-06
*USP17L10*	41	12	1.55E-05
*ZFPM1*	70	23	1.12E-07
*ZNF211*	55	16	5.14E-07
*ZNF598*	70	12	5.53E-12

We included only genes where the difference between affected cases and controls have a p value <4.47E-05. P values are Bonferroni-corrected significantly different affected genes. From a total of 1,119 genes with high impact variants in both cases and controls, 60 official gene names were identified as harboring high impact mutations in cases and controls. Within this list of differentially affected genes 12/60 (20%) are T cell receptors, shown ordered alphabetically for easier interpretation.

### Functional enrichment analysis

The resulting differently affected 60 genes were analyzed for functional enrichment using DAVID.^24^^,^^25^ We used DAVID’s functional annotation chart output to cluster groups of genes according to their enriched term score. In Table S1, we provide the non-redundant set of terms below the significance threshold and their associated genes, ordered by the strength of their statistical significance.

**Figure 4:**
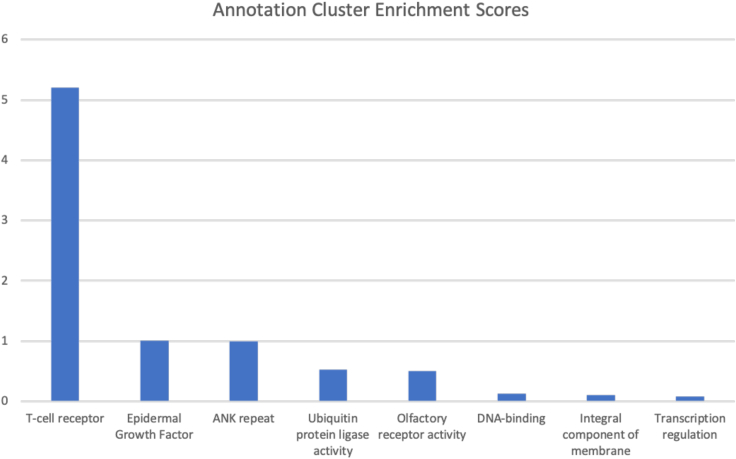
Gene cluster enrichment annotations Results from functional enrichment analysis of top affected genes. The T cell receptor cluster enrichment score (DAVID Enrichment Score: 5.21) is the top scoring functional cluster, followed by epidermal growth factor (1.00) and ANK repeat (0.99).

A cluster of genes related to T cell receptors dominated the DAVID’s output table. The T cell receptor cluster enrichment score (Enrichment Score: 5.21) is the top scoring functional cluster, followed by epidermal growth factor (1.00) and ANK repeat (0.99). Figure 4 shows DAVID’s enrichment scores for term clusters resulting from analyzing those 60 genes.

### Analysis of TCR gene cluster variants

Next, we focused on the analysis of high impact variants within genes of the top functionally enriched cluster. This analysis yielded 25 variants with case-control allele frequency differences below a threshold of genome-wide significance (p value <5.0E-08; Table 4). These variants were distributed among 8 of the 12 T cell receptor gene cluster and included *TRBV7-8*, *TRBV7-7*, *TRBV7-6*, *TRBV5-5*, *TRBV6-5*, *TRBV10-1*, *TRBV7-3*, and *TRBV30*. All variants are relatively common (> 0.01 within the European population; NCBI’s ALFA Allele Frequency Aggregator^26^). These 25 variants include 4 stop gains (single nucleotide variant substitution) and 21 frameshifts (indels), all of them highly deleterious according to CADD^27^ and localized within the TRB locus on chromosome 7, at band 34 within the long arm (7q34). We did not filter these variants by linkage disequilibrium, given that they are all functional (consequence either frameshift variant or stop gained).

**Table 4:** Variants of concern in T cell receptor beta variable genes

GENE	RSID	Chr	Chr Start	ChrEnd	REF	ALT	Consequence	CADD PHRED	Case alleles count	Control alleles count	Case MAF	Control MAF	Case Control P-Value	EUR Sample Size	EUR MAF	Case EUR P-Value
*TRBV7-8*	rs752460700	7	142099588	142099588	C	CACTG	frameshift_variant	19.1	52	1	0.351	0.005	2.62629E-15	10574	0.038	2.832E-169
	rs758031370	7	142099590	142099590	T	TG	frameshift_variant	23.1	52	1	0.351	0.005	2.62629E-15	10574	0.037	5.6577E-171
	rs763698275	7	142099593	142099595	TTC	T	frameshift_variant	15.4	35	1	0.236	0.005	1.45773E-10	10574	0.029	9.6133E-101
*TRBV7-7*	rs1463284969	7	142119876	142119877	CG	C	frameshift_variant	16.4	74	2	0.500	0.011	9.75076E-21	11474	0.134	1.30317E-87
	rs1461692463	7	142119879	142119879	C	CCA	frameshift_variant	23.5	74	3	0.500	0.016	4.42527E-20	4494	0.342	3.25993E-21
	rs1163453604	7	142119881	142119882	GA	G	frameshift_variant	22.2	74	3	0.500	0.016	4.42527E-20	14202	0.109	3.1952E-114
*TRBV7-6*	rs1414820805	7	142139334	142139335	CG	C	frameshift_variant	16.9	74	14	0.500	0.075	4.40839E-14	20263	0.018	>5.0E-200
	rs1440315119	7	142139337	142139337	C	CCA	frameshift_variant	25.0	74	14	0.500	0.075	4.40839E-14	4512	0.085	9.8566E-135
	rs1180782962	7	142139339	142139340	GA	G	frameshift_variant	21.6	74	14	0.500	0.075	4.40839E-14	4512	0.088	5.4848E-131
*TRBV5-5*	rs747286228	7	142148969	142148969	A	T	stop_gained	23.2	74	25	0.500	0.134	8.26204E-10	18192	0.235	3.0043E-41
*TRBV6-5*	rs373875376	7	142180584	142180586	TCC	T	frameshift_variant	22.7	74	21	0.500	0.113	3.34905E-11	10574	0.340	5.21652E-22
	rs775454437	7	142180591	142180593	TGG	T	frameshift_variant	22.3	74	21	0.500	0.113	3.34905E-11	17536	0.204	4.49808E-51
	rs761774365	7	142180593	142180593	G	GTTTT	frameshift_variant	22.8	74	21	0.500	0.113	3.34905E-11	10574	0.338	3.15424E-22
*TRBV10-1*	rs17249	7	142231625	142231625	C	A	stop_gained	35.0	71	27	0.480	0.145	1.5867E-08	30772	0.457	1.77686E-10
	rs999255927	7	142231780	142231780	C	T	stop_gained	29.5	74	24	0.500	0.129	3.84216E-10	4512	0.485	3.71185E-10
	rs1395412038	7	142231793	142231793	A	AT	frameshift_variant	22.8	74	22	0.500	0.118	7.74139E-11	4512	0.483	2.9271E-10
	rs1364171206	7	142231796	142231798	CCA	C	frameshift_variant	22.7	74	22	0.500	0.118	7.74139E-11	4512	0.481	2.56595E-10
	rs1215288016	7	142231800	142231800	G	GA	frameshift_variant	22.1	74	21	0.500	0.113	3.34905E-11	4512	0.478	1.72077E-10
	rs1296143203	7	142231805	142231805	C	CTG	frameshift_variant	16.0	74	21	0.500	0.113	3.34905E-11	4512	0.476	1.34619E-10
	rs1216496208	7	142231808	142231810	GCC	G	frameshift_variant	18.3	74	20	0.500	0.108	1.41216E-11	4512	0.472	7.6761E-11
*TRBV7-3*	rs764426432	7	142247529	142247531	GGC	G	frameshift_variant	21.4	74	17	0.500	0.091	8.99976E-13	4512	0.482	2.69204E-10
	rs751581456	7	142247535	142247535	G	GAA	frameshift_variant	20.6	74	17	0.500	0.091	8.99976E-13	4508	0.485	3.80278E-10
	rs757429570	7	142247538	142247540	CTG	C	frameshift_variant	12.0	73	10	0.493	0.054	9.21051E-16	4512	0.305	5.06241E-25
	rs781428044	7	142247541	142247541	C	CAT	frameshift_variant	19.5	73	11	0.493	0.059	2.96063E-15	4512	0.309	1.61172E-24
*TRBV30*	rs17267	7	142510446	142510446	G	A	stop_gained	38.0	52	11	0.351	0.059	8.27473E-10	33888	0.214	1.39201E-19

A total of 25 variants in GRCh37 were identified in 8 T cell receptor beta variable genes below genome-wide threshold of significance (p value <5.0E-08) for allele frequency differences in cases and controls. All variants cluster within a region ∼0.5M nucleotides long in chromosome 7 at band 7q34. 21 of them are indels causing frameshift mutations, the rest single nucleotide stop gain variants. All of them are highly deleterious according to CADD. A CASE_MAF = 0.500 means all cases are heterozygous for the alternative allele. Case control p value relates to chi squared statistical significance difference between allele frequencies in our population of cases and controls. Case EUR p value relates to chi squared test differences between frequencies in the general European (EUR Sample Size) from NCBI’s ALFA allele frequencies and the case population (Abbreviations: RSID = dbSNP ID; REF = Reference allele; ALT = Alternative allele; CADD_PHRED = Combined Annotation Dependent Depletion Phred Score; MAF = Minor Allele Frequency; EUR_MAF = NCBI’s ALFA Allele Frequency Aggregator for the alternative allele).

### TCR loss of function via compound heterozygosis

Except for a few exceptions, most of the 25 variants of concern were heterozygous for the alternative allele in our patients. In order to test whether loss of function might occur in both alleles in the remaining 8 T cell receptor genes, we counted high impact alleles within each of them (*TRBV5-5*, *TRBV6-5*, *TRBV7-3*, *TRBV7-6*, *TRBV7-7*, *TRBV7-8*, *TRBV10-1*, *TRBV30*). If a gene harbors more than 1 high impact allele, the chances for compound heterozygosis are greater and therefore the chances for gene inactivation. Because of the limitations of short read sequencing, which does not distinguish phase between alleles in different variants, it still may be possible for two heterozygous high impact variants to affect the same allele. As a consequence, we decided to classify as likely compound heterozygous loss of function the presence of at least *three* high impact alleles in a TCR. Table 5 shows counts of high impact alleles per patient for each of the TCR. For each patient we therefore counted the number of high impact mutations within variants that already have been identified with significant allele frequencies in cases and controls. From a total of 25 variants of concern spanning 8 T cell receptor beta variable (TRBV) genes, we found these three groups.

1. TRBV genes with likely inactivation through compound heterozygosis: mutations in all patients ranging from 3 to 8 (*TRBV6-5*, *TRBV7-3*, *TRBV7-6*, *TRBV7-7*, *TRBV10-1*). There is an exception in *TRBV7-3*, with 1 patient having only two alleles.
2. TRBV genes with some patients showing potential for inactivation via compound heterozygosis (*TRBV7-8*, *TRBV30*). High impact alleles are either absent or up to 3.
3. TRBV genes unlikely to be inactivated through compound heterozygosis (*TRBV5-5*). High impact alleles never make it to 2 for any patient.

**Table 5:** T cell receptor beta variable (TRBV) genes with significantly different allele frequency variants between cases and controls

TCR GENES	TRBV5_5	TRBV6_5	TRBV7_3	TRBV7_6	TRBV7_7	TRBV7_8	TRBV10_1	TRBV30
#Variants of Concern in Gene	1	3	4	3	3	3	7	1
PATIENT ID	#HI Alleles	#HI Alleles	#HI Alleles	#HI Alleles	#HI Alleles	#HI Alleles	#HI Alleles	#HI Alleles
AR5440	1	3	4	3	3	2	7	2
AR5443	1	3	4	3	3	3	8	0
AR5444	1	3	4	3	3	3	6	0
AR5445	1	3	4	3	3	0	6	1
AR5446	1	3	4	3	3	3	8	1
AR5447	1	3	4	3	3	3	7	0
AR5448	1	3	4	3	3	2	7	0
AR5449	1	3	4	3	3	2	6	1
AR5450	1	3	4	3	3	3	7	2
AR5451	1	3	4	3	3	0	7	0
AR5452	1	3	4	3	3	0	6	2
AR5454	1	3	4	3	3	3	7	0
AR5455	1	3	4	3	3	0	7	0
AR5457	1	3	4	3	3	2	7	0
AR5458	1	3	4	3	3	3	6	2
AR5459	1	3	4	3	3	3	8	0
AR5460	1	3	4	3	3	2	7	0
AR5461	1	3	4	3	3	0	7	0
AR5462	1	3	4	3	3	3	6	1
AR5463	1	3	4	3	3	3	7	0
AR5464	1	3	4	3	3	0	6	1
AR5465	1	3	2	3	3	0	7	2
AR5466	1	3	4	3	3	3	7	1
AR5467	1	3	4	3	3	2	7	0
AR5468	1	3	4	3	3	2	7	2
AR5469	1	3	4	3	3	3	8	0
AR5470	1	3	4	3	3	3	7	1
AR5472	1	3	4	3	3	3	6	0
AR5473	1	3	4	3	3	3	7	1
AR5474	1	3	4	3	3	0	8	0
AR5475	1	3	4	3	3	0	7	1
AR5476	1	3	4	3	3	0	8	0
AR5477	1	3	4	3	3	3	8	0
AR5478	1	3	4	3	3	0	7	1
AR5481	1	3	4	3	3	0	8	1
AR5484	1	3	4	3	3	0	7	0
AR5485	1	3	4	3	3	0	6	0
AR5486	1	3	4	3	3	3	6	0
AR5487	1	3	4	3	3	0	7	1
AR5488	1	3	4	3	3	3	7	2
AR5490	1	3	4	3	3	0	6	0
AR5492	1	3	4	3	3	3	7	0
AR5493	1	3	4	3	3	3	7	2
AR5495	1	3	4	3	3	2	6	1
AR5496	1	3	4	3	3	3	7	1
AR5497	1	3	4	3	3	3	6	0
AR5499	1	3	4	3	3	2	7	0
AR5500	1	3	4	3	3	2	8	1
AR5501	1	3	4	3	3	0	6	0
AR5502	1	3	4	3	3	3	7	2
AR5503	1	3	4	3	3	0	7	0
AR5506	1	3	4	3	3	3	7	1
AR5507	1	3	4	3	3	0	7	1
AR5508	1	3	4	3	3	3	6	1
AR5510	1	3	4	3	3	3	7	1
AR5511	1	3	4	3	3	3	8	0
AR5512	1	3	4	3	3	0	8	0
AR5513	1	3	4	3	3	0	8	1
AR5514	1	3	4	3	3	3	8	1
AR5516	1	3	4	3	3	2	7	0
AR5517	1	3	4	3	3	3	6	2
AR5518	1	3	4	3	3	2	7	1
AR5520	1	3	4	3	3	3	7	1
AR5521	1	3	4	3	3	3	6	1
AR5522	1	3	4	3	3	2	7	1
AR5524	1	3	4	3	3	3	7	2
AR5526	1	3	4	3	3	2	7	1
AR5527	1	3	4	3	3	2	8	0
AR5530	1	3	4	3	3	2	7	1
AR5533	1	3	4	3	3	2	6	0
AR5535	1	3	4	3	3	3	8	1
AR5536	1	3	4	3	3	0	8	2
AR5538	1	3	4	3	3	3	7	0
AR5539	1	3	4	3	3	3	6	0

For each gene we highlight the total number of variants of concern as well as the number of high impact alleles each patient has in each gene for those variants. In red we highlight those genes containing more than 3 high impact alleles, indicating a potential loss of function in both alleles for the gene.

In a preliminary analysis, we could not find any exome sequencing reads flanking the TRBV genes that could suggest a recombination with D genes (data are not shown).

### Comparison with genetic markers previously reported as determinants of severe COVID-19

To date a number of genetic variants have been associated to COVID-19 disease severity. None of them, however, have implicated T cell receptor genes. Table 6 shows the latest list of variants from the Genetics of Mortality in Critical Care (GenOMICC) study that are present in our patient cohort.^8^ We chose only variants from the GenOMICC study because it involved severe COVID-19 patients and were produced using whole genome sequencing data. For 23 lead variants from the GenOMICC study, our exome patient data covered 11 of them with sufficient quality (STAR Methods). We calculated allele risk frequencies in our filtered case cohort (N = 74) and compared them to risk allele frequencies in NCBI’s ALFA Europeans. We found no significant differences between allele risk frequencies in our filtered case cohort compared to the European population (ρ = 0.9708; p value = 0.7327).

**Table 6:** Lead variants from the GenOMICC study and their frequencies in our filtered case cohort of 74 IBS cases (IBS COV AF) with severe COVID-19

RSID	Gene	Chromosome	Chr_start (GRCh37)	Chr_end (GRCh37)	Risk Allele	Odds Ratio	Total Alleles	Risk Allele Count	IBS COV AF (n = 74)	EUR Sample Size	EUR MAF
rs114301457	*EFNA4*	1	155039464	155039464	T	2.4	148	0	0	37,196	0.00519
rs7528026	*TRIM46*	1	155147781	155147781	A	1.4	142	9	0.0634	14,336	0.02741
rs41264915	*THBS3*	1	155167786	155167786	A	1.3	148	140	0.9459	30,840	0.89066
rs2271616	*SLC6A20*	3	45838013	45838013	T	1.3	148	21	0.1419	16,692	0.12197
rs343320	*PLSCR1*	3	146234909	146234909	A	1.2	148	10	0.0676	246,424	0.081871
rs28368148	*IFNA10*	9	21206605	21206605	G	1.7	148	2	0.0135	21,546	0.01109
rs61882275	*ELF5*	11	34504292	34504292	G	1.1	22	22	1	14,370	0.63319
rs117169628	*SLC22A31*	16	89262657	89262657	A	1.2	148	13	0.0878	22,920	0.11898
rs12610495	*DPP9*	19	4717672	4717672	G	1.3	148	57	0.3851	171,196	0.283651
rs73510898	*ZGLP1*	19	10416444	10416444	A	1.3	144	11	0.0764	29,172	0.06664
rs34536443	*TYK2*	19	10463118	10463118	C	1.5	148	6	0.0405	98,022	0.04200

From a total of 23 variants in GenOMICC, our exome data covered 11 variants (shown here). We calculated risk allele frequencies in our case cohort and compared them to risk allele frequencies in Europeans from NCBI’s ALPHA Allele Frequencies. MAF counts can be inferred from EUR sample sizes and their respective EUR MAFs.

We looked into the Host Genetics Initiative for COVID-19 as a public independent dataset.^28^ We chose the subset of patients with very severe COVID-19. This dataset included sequencing and microarray variant data from 21 studies across distinct European populations. We downloaded this dataset in GRCh37 (A2_ALL_eur_leave_23andme). From our total of our 25 variants of concern recorded in Table 6, we identified two that were included in the study. These two variants were rs17249 (7:142400325:G:T; Reverse Complemented Alleles) and rs17267 (7:142812761:G:A). Both variants are stop codons and have allele frequencies of 0.4526 and 0.239, respectively, which is similar to the European allele frequencies in the NCBI ALFA controls in Table 6. We found that both alternative alleles for these two variants are present in all our cases (n = 74) in heterozygosis, which suggests a greater frequency than the one noticed by the HGI consortium. No other variants from our dataset were present in this subset of Europeans with severe COVID-19. The lack of presence of most of our variants could be due to differences in methodology and study population. Our methodology used exome sequencing from highly selected Iberian Spanish (IBS) cases and controls, bioinformatically processed in the same way. We did not use a meta analysis of microarray, exome and whole genome studies, which implies different filtering and quality controls. For instance, we did not apply a linkage disequilibrium filter, given that we only considered coding variants with high impact, being either frameshifts of stop codons. We included both rare and common variants in our analysis, with most of our variants being multinucleotide insertions or deletions, and not SNPs as it is the case within the HGI dataset.

## Discussion

After performing whole exome sequencing of a selected sample of IBS patients with severe COVID-19, we found a group of TCR chain encoding genes more likely to be inactivated in our patient cohort. Our study identified 25 high impact heterozygous variants at the T cell receptor beta variable (TRBV) locus on human chromosome 7. Twenty of these variants were present in most patients suggesting likely TRBV inactivation via compound heterozygosis of the following 5 genes: *TRBV6-5*, *TRBV7-3*, *TRBV7-6*, *TRBV7-7*, *TRBV10-1*. These genes are all part of the TCR beta complex, participating in highly specific antigen recognition. Altogether our findings support that a genetic predisposition may account for suboptimal and dysfunctional T cell responses in SARS-CoV-2 infection might favor the development of severe COVID-19.

A striking feature of SARS-CoV-2 infection is that it may produce a wide range of symptoms, from asymptomatic infections to acute respiratory distress syndrome. Other complications include thromboembolic phenomena and clinical manifestations due to specific organ involvement (hepatitis, renal failure, cardiovascular events, neurological dysfunction, etc.).^29^ Although distinct inoculum sizes^30^ and different coronavirus variants may determine differences in transmission and pathogenicity,^31^ host factors seem to largely explain the wide range of clinical outcomes seen following SARS-CoV-2 infection. Among others, older age, male sex and the presence of comorbidities (obesity, diabetes, prior lung disease, immunosuppression, etc.) are well-established predictors of severe COVID-19.^34^ Our data corroborate a predominance of male individuals among those with severe COVID-19 consecutively attended during the first wave of COVID-19 in Madrid, Spain. For facilitating the search of host genetic determinants, older individuals and those with comorbidities were excluded from our study cohort.

Our analysis used a set of matched ancestry case-control individuals whose exome data were processed and filtered using the same protocol. Variant data were analyzed using VEP, in order to discover high impact mutation (likely loss of function) count differences in cases and controls.

In order to minimize biases and artifacts for observed differences in severe COVID-19 patient-affected genes, we followed a strict set of filters, both at the level of variant and sample selection. Our genetic study targeted specifically a subset of apparently healthy individuals younger than 60 years-old that developed severe COVID-19 and required hospitalization. We found a significant enrichment in loss of function at the TRB locus on the long arm of chromosome 7, at band 7q34. Recent reports from the GenOMICC uncovered seven risk genes associated with severe COVID-19 infection located on chromosomes 6 (nearby where the HLA system lies in humans), 12, 19, and 21.^8^ Other studies have investigated genetic determinants of severe COVID-19 in a much broader clinical population. In many of these studies, genes that mediate immune responses have been found, particularly those clustering a region at chromosome 3 and others mediating interferon responses.^5^^,^^6^^,^^7^^,^^8^^,^^9^^,^^10^^,^^11^^,^^12^^,^^13^ However, heterogeneity in ancestry study populations, clinical definition criteria, and methodological issues have resulted in lack of uniform findings and recognition of overall impact of genetic markers on COVID-19 disease severity.^32^

Our results show the power of highly selective inclusion clinical criteria, together with the importance of selecting for high impact variants and clustering of variants according to their annotated functions. Our method for variant selection and gene clustering allowed us to find enrichment for loss of function in TCR genes. These are a class of T cell surface molecules that recognize the antigen-derived peptides presented by the MHC and are able to trigger a series of immune responses. Variants identified in our study suggest a mechanism for T cell dysfunction/extenuation that could lead to severe COVID-19. There is evidence of terminally differentiated T cells or possibly exhausted T cells in severe disease, with increased expression levels of the inhibitory receptors *PD1*, *TIM3*, *LAG3*, *CTLA4*, *NKG2A*, and *CD39.*^33^^,^^34^^,^^35^^,^^36^^,^^37^^,^^38^ Nevertheless, expression of these receptors could also reflect T cell activation. Our data provide evidence for a suboptimal or otherwise inappropriate T cell response associated with severe COVID-19.^39^

We expect our findings might help prioritize patients more likely to suffer from severe COVID-19 as carriers of these genetic determinants. Our results contribute to the much wider debate on the importance of analyses of diverse human ethnic groups, since we have only used Iberian Spanish (IBS) patients to draw these results. Similar analyses in different populations will be therefore needed with a greater number of patients and controls. In summary, we propose a crucial role of T cell receptor genes as determinants of severe COVID-19. Our findings deserve further consideration by better powered studies and in distinct ethnic groups.

### TCR functional gene cluster significantly enriched

A total of 60 genes were identified as having significantly different counts of high impact mutations. Overall, 12 out of these 60 genes were TCR genes. Functional clustering analysis within these 60 genes confirmed the TCR beta gene cluster to be far more enriched than any other. Apart from TCR, some of the remaining genes are evolutionarily related and already known to influence COVID-19 severity, such as genes of mucin secretion (*MUC5B*)^40^ or the *GOLGA6L2* family.^41^

### New variant associations for T cell receptor beta variable (TRBV) genes

Functional enrichment analysis led us to analyze variants within the TRBV gene cluster. Overall, 25 high impact variants of concern spanned eight TRBV beta encoding genes (*TRBV5-5*, *TRBV6-5*, *TRBV7-3*, *TRBV7-6*, *TRBV7-7*, *TRBV7-8*, *TRBV10-1*, *TRBV30*) displaying genome wide significant (p < 5.0E-08) allele frequency differences. All of these variants are common (≥0.236 frequency) in our case cohort, and also common (≥ 0.018) in the European population, according to the NCBI’s ALFA. Our relatively common variants of concern are compatible with the ∼10% adult population who contract severe COVID-19 disease. Allele frequencies of these variants appear significantly different in our case cohort compared to the European population (p value = 5.29E-06; ANOVA Single Factor), which suggests an enrichment of their frequency for our cases with respect to Europeans. All of these variants are part of the hypervariant TCR V region of beta chains, yet they all are relatively common and highly deleterious (CADD Phred score =>12.0).

Almost all observed high impact alleles from these 25 variants were heterozygous. If a T cell uses a non-productive TCR, it would therefore be free to rearrange another TRBV. If a particular TRBV is not available as it is the case in half of the alleles that have one of the variants, the T cell may arrange the normal allele on the other chromosome. If we consider heterozygous high impact mutations in isolation, it is therefore likely that the repertoire will not be affected unless the other chromosome gene copy contains another high impact mutation from a different variant. The presence of two different mutated alleles at a particular locus can inactivate a gene, a process which is known as compound heterozygosis. To ascertain whether compound heterozygosis could be present for each of these 8 TCRBV genes, we counted high impact alleles within the same patient. We analyzed how many high impact alleles patients had within the 25 variants of concern. We identified three groups of genes: (a) 5 TRBV genes where compound heterozygosis was likely for all patients because they harbored more than 3 high impact alleles (*TRBV6-5*, *TRBV7-3*, *TRBV7-6*, *TRBV7-7*, *TRBV10-1*); (b) two where compound heterozygosis was possible for some patients with 0–3 mutant alleles (*TRBV7-8*, *TRBV30*), and (c) one gene where no compound heterozygosis was possible (*TRBV5-5*).

Because sequencing is unable to determine the phase of high impact alleles, we expect higher chances for high impact mutations affecting both chromosomal copies with greater high impact alleles. More than 3 high impact alleles were counted for almost all patients in five genes (*TRBV6-5*, *TRBV7-3*, *TRBV7-6*, *TRBV7-7*, *TRBV10-1*), suggesting their possible inactivation. Such inactivation of TRBV could lead to reduced repertoire or poorer specific T cell activation for our 74 severe patients. A less specific immune response would result in a dysfunctional activation with a much broader cytokine and inflammatory systemic response.

### The role of TCR in patients with severe COVID-19

To date, the role of TCR in COVID-19 severity has remained unclear. Some studies^42^^,^^43^^,^^44^ have shown that T cells play a prominent role in COVID-19 susceptibility and severity. However, they have not been able to establish whether T cell responses are helpful or harmful. Prominent lymphopenia has been observed in patients with severe disease, with abnormal T cell differentiation.^45^^,^^46^ Moreover, reduction of T cells in the periphery is a prominent feature of many individuals with severe COVID-19. Given the high impact of the identified variants, our results support that loss of function and inactivation of T cell receptors affecting the variable region in charge of binding to the peptide/MHC complex as a genetic signature for severe COVID-19. TCR may therefore play a crucial role in the recognition of SARS-CoV-2 antigens by T cells, accounting for a dysfunctional response for an exacerbation of symptoms in SARS-CoV-2 infection.

### Risk allele frequencies do not differ from the general European population

We compared existing published genome-wide association variants from the GenOMICC consortium, which were unveiled in a large population of mostly Northern European individuals. The GenOMICC study provides a state-of-the-art analysis of host genomics associated with disease severity, yielding 23 genome wide significant variants. From this list, 11 were covered with sufficient quality in our 74-case cohort. We then compared risk allele frequencies observed in our case cohort against those of the general European population, yielding no significant differences. The lack of significant differences in our case/control cohorts for GenOMICC risk alleles may be due to their small sample, the peculiar characteristics of IBS ancestry or the different methodology we used. Our frequency concordance with the general European population, however, supports the validity of our variant frequency data, which, although small, follow the expected patterns observed for the European population in an independent cohort (NCBI’s ALFA).

### Limitations of the study

We acknowledge the modest size of our study population. We prioritized the use of strict clinical criteria to define severe COVID-19 in addition to checking a restricted ancestry-matched population. We also note that individuals used as controls in our study were from the general population rather than confirmed SARS-CoV-2 infected individuals with no symptoms. This means that a very small proportion of our controls could also be liable to suffer severe COVID-19 following coronavirus infection. This is also reflected by design, where all high impact variants in T cell receptors are present in cases and controls, albeit with significantly different frequencies. Despite this, our general population controls allow us sufficient discriminatory power to statistically identify differences in affected genes when comparing cases and controls.

## STAR★Methods

### Key resources table

**Table:** 

REAGENT or RESOURCE	SOURCE	IDENTIFIER
**Deposited data**		

Raw and processed data	This paper	EGA: EGAC00001002480
IBS data	1000 Genomes Project	https://www.internationalgenome.org
Human reference genome NCBI build 37, GRCh37	Genome Reference Consortium	http://www.ncbi.nlm.nih.gov/projects/genome/assembly/grc/human/

**Software and algorithms**		

BWA-MEM	Heng Li^46^	https://doi.org/10.48550/arXiv.1303.3997
GATK4	Heldenbrand, J. R. et al.^43^	https://gatk.broadinstitute.org/hc/en-us/articles/360036194592-Getting-started-with-GATK4
PLINK	Purcell, S. et al.^48^	https://www.cog-genomics.org/plink/
bcftools	Danecek, P. et al.^49^	https://samtools.github.io/bcftools/bcftools.html
Variant Effect Predictor	McLaren, W. et al.^50^	https://www.ensembl.org/info/docs/tools/vep/index.html
Sift	Sim, N.-L. et al.^52^	https://www.ensembl.org/info/docs/tools/vep/index.html
PolyPhen	Adzhubei, I. et al.^53^	https://www.ensembl.org/info/docs/tools/vep/index.html
CADD	Rentzsch, P. et al.^54^	https://www.ensembl.org/info/docs/tools/vep/index.html
Condel	González-Pérez, A. & López-Bigas, N^55^	https://www.ensembl.org/info/docs/tools/vep/index.html
LoFtool	Fadista, J. et al.^56^	https://www.ensembl.org/info/docs/tools/vep/index.html
MPC	Samocha, K. E. et al.^57^	https://www.ensembl.org/info/docs/tools/vep/index.html
DAVID	Huang, D. W. et al.^60^	http://david.abcc.ncifcrf.gov

**Other**		

Best practices for variant discovery analysis	Broad Institute	https://gatk.broadinstitute.org/hc/en-us/sections/360007226651-Best-Practices-Workflows
Best practices for variant discovery analysis and quality control	NYU Center for Genomics and Systems Biology (CGSB)	https://gencore.bio.nyu.edu/variant-calling-pipeline-gatk4/
1000G_phase1.indels.b37.vcf	1000 Genomes Phase I indel calls	https://gatk.broadinstitute.org/hc/en-us/articles/360035890811-Resource-bundle
1000G_phase3_v4_20130502.sites.vcf	1000 Genomes Phase 3 indel calls	https://www.internationalgenome.org/category/phase-3/
gnomAD	Whiffin, N. et al.^51^	https://gnomad.broadinstitute.org
ALFA: Allele Frequency Aggregator	NCBI	https://www.ncbi.nlm.nih.gov/snp/docs/gsr/alfa/

### Resource availability

#### Lead contact

Further information and requests for resources should be directed to and will be fulfilled by the lead contact, Manuel Corpas (m.corpas@westminster.ac.uk).

#### Materials and availability

No new materials have been created as part of this manuscript.

### Experimental model and study participant details

#### Consent for publication

In compliance with the provisions of the Declaration of Helsinki and the legislation in force in Spain regarding research with human beings, patients were informed about their participation in this clinical study, clarifying that their participation was voluntary and did not imply any change in his/her treatment or medical care compared to what s/he would receive if s/he did not participate. All patient informed and voluntary consents were obtained in writing (see supplemental information for consent forms and patient data collected).

#### Ethics approval

This study was evaluated and approved by the Clinical Research Ethics Committee of Hospital Clínico San Carlos (code number: 20/313-E_COVID) in Madrid, Spain.

#### Study population

We retrospectively identified all consecutive individuals hospitalized at one large tertiary hospital in Madrid during the first wave of COVID-19 with confirmed SARS-CoV-2 infection and at least the following five clinical features: i) age younger than 60 years-old; ii) fever and respiratory symptoms for more than 3 days; iii) arterial oxygen saturation below 93%; iv) bilateral pneumonia on imaging techniques; and v) absence of comorbidities such as diabetes, obesity, immunosuppressive conditions, etc. This restrictive definition of severe COVID-19 resembles that of the earlier Chinese studies lately adopted by the NIH.^29^ All patients were non-vaccinated at the time of sample collection.

#### Patient Clinical History

We annotated age, sex, country of origin, date of symptoms initiation, date for hospitalization and discharge, and whether there was admission to intensive care unit (ICU). We also recorded body mass index, smoking status, and whether patients had hypertension, diabetes mellitus, or other relevant medical conditions. Laboratory, radiology and information on treatment interventions was similarly recorded.

Our initial cohort of 98 cases included 33 women and 65 men. All patients were hospitalized and 22 of them needed intensive care unit (ICU). The mean patient age was 51.1 years old. By sexes, the female average age was 49.0 and male 52.1 years old. The average stay in hospital for all patients was 20.7 days while for females 12.2 and males 25.0 days. For the subset of 22 patients who stayed at the ICU, the average stay was 26.2 days while for females only it was 7.2 days while for males 30.4 days on average.

#### Clinical phenotypic characterization

We developed our own controlled vocabulary in order to describe in detail COVID-19 clinical phenotypes in our patients. Although we considered using other COVID-19 phenotype ontologies, such as those offered by CIDO^47^ or HPO^48^ our need for further granular detail prompted us to develop our own. We therefore developed 28 medical terms grouped into 4 major clinical phenotypes, which were then checked for their presence in each patient.

#### Case/control cohort

Whole blood was stored for all hospitalized COVID-19 patients as part of a larger study conducted by the hospital research unit. Severe illness and intensive care unit (ICU) admission were recognized for 98 and 22 patients, respectively. For controls, we downloaded exome raw FASTQ files from all available exomes within the Iberian Spanish (IBS) subpopulation of the 1000 Genomes Project (1000G). All exomes (205) were bioinformatically processed in an identical manner as shown below.

### Method details

#### Bioinformatics workflow

Figure 2 illustrates an overview of the study workflow. The details of how we performed each of the steps are described in detail below. Whole exome sequencing was performed for 100 selected cases. Bioinformatics processing for these 100 cases was also performed for the 1000 Genomes Project (1000G) exome data from the Iberian Spanish (IBS) subpopulation (n=107). A principal component analysis (PCA) was then carried out to select from the 100 cases those that clustered within the IBS 1000G subpopulation. 74 cases were selected for further analysis. To control for batch effects, we only considered QC’d variants present in both cases and controls. Next, their protein effect was predicted. We performed a gene-based collapsing analysis, where we counted the number of cases and controls with high impact variants per gene. Genes above a threshold of significant difference in cases and controls were then clustered using DAVID. High impact variants within genes from the TCR cluster were then analyzed.

#### DNA extraction, library construction and exome sequencing of cases

A total of 100 blood samples were collected in 10 mL EDTA tubes. All samples were centrifuged at 3000 rpm for 10’ and a buffy coat was isolated and frozen at -20°C until their use. Genomic DNA was isolated from buffy coat frozen samples with the Maxwell RSC buffy coat DNA kit (Promega) using the Maxwell RSC Instrument (Promega), following manufacturer recommendations. After isolation, the purity of genomic DNA was analyzed by spectrometric analysis. Whole exome sequencing was performed using ExomeCapture-Seq capture KAPA HyperExome on Illumina machines.

#### Genome alignment and variant generation

We generated variant calls for cases and controls using identical parameters. Our pipeline used GATK4^49^ based on best practices for variant discovery analysis from the Broad Institute^50^ and the NYU Center for Genomics and Systems Biology (CGSB).^51^ BWA-MEM^52^ was used to align all reads to GRCh37.

#### Genotyping, sample selection, and variant quality control summary

We performed quality control (QC) as indicated by the NYU Center for Genomics and Systems Biology (CGSB).^51^ Namely, from 716 initial FASTQ files, we produced 100 BAM files, one for each of the samples sequenced. We noted that sample AR5463 had a premature end of file, which we discarded. Read duplicates were marked and alignment metrics and insert size metrics generated using Picard’s validation stringency parameters as strict. All the reads in BAM files were then merged to a single new read-group. Base Quality Score Recalibration (BQSR) was performed with a ‘gold standard’ set of indels and SNPs. These gold standard indels included 1000G_phase1.indels.b37.vcf (currently from the 1000 Genomes Phase I indel calls), Mills_and_1000G_gold_standard.indels.b37.sites.vcf and the latest set from 1000G phase 3 (v4) for genotype refinement from 1000G_phase3_v4_20130502.sites.vcf.^53^ The recalibration was applied to all BAM files. Indels and SNPs were processed and then filtered independently using parameters provided by the Broad Institute. For SNPs we used QD < 2.0, FS > 60.0, MQ < 40.0, SOR > 4.0, MQRankSum < -8.0. For indels, QD < 2.0, FS > 200.0 and SOR > 10.0. It was found that one sample was in triplicate. We therefore eliminated two of the three samples moving forwards. For controls, 14 samples were discarded due to corrupted or premature end of FASTQ files. 98 cases and 93 controls successfully completed our alignment and variant call pipelines after QC. PCA analysis confirmed that the remaining samples for analysis were not relatives.

#### Case sample selection

A total of 98 hospitalized patients with severe COVID-19, were identified using the inclusion criteria described above (cases) and sample QC criteria. Although all cases were sampled in Madrid, a significant number (19.4%) of individuals had been born outside of Spain (Figure 3).

We performed a principal component (PC) analysis using PLINK^54^ and R to select only cases whose ancestry directly matched that of 1000G controls, selecting for further analysis only cases whose principal components clustered within the cluster of IBS 1000G controls. In order to check the genetic ancestry of all samples, we compared them against all 1000G individuals (Figure S1), showing that the vast majority of patients clustered, as expected, within the 1000G European continent.

In order to eliminate the potential bias due to ancestral differences, we defined controls as exomes from Iberian Spanish (IBS) ancestry in the 1000G that successfully completed our pipelines and quality controls (N=93). We then used principal component analysis to filter out cases clustering outside of IBS controls. For that purpose, we used the first (PC1) and second principal components (PC2), selecting only those cases whose PC1 and PC2 were > than the lowest of the controls, and < than the highest (Figure S2). This left us with 74 cases for further analysis (22 female and 52 male).

#### Variant filtering

We concatenated indels and single nucleotide variant (SNV) files and merged them via bcftools.^55^ All variants were further filtered by quality of the call (QUAL>20) and read depth (DP>10). We also kept variants within autosomes, filtering out variants in the sexual chromosome pair and mitochondria.

#### Variant effect prediction

The remaining variants were analyzed using Variant Effect Predictor (VEP).^23^ All analyses were configured via VEP’s interface to include gene symbols, 1000 Genomes global minor allele frequency, gnomAD allele frequencies^56^ and all computational pathogenicity predictions (Sift,^57^ Polyphen,^58^ CADD,^27^ Condel (prediction + score),^59^ LoFtool^60^ and MPC^61^). We applied no VEP filtering other than selecting 1 consequence per variant. For prioritization and selection of affected genes, we only kept those variants predicted by VEP as ‘high impact’ (i.e., the variant is assumed to have high (disruptive) impact in the protein, probably causing protein truncation, loss of function or triggering nonsense mediated decay).^62^

### Quantification and statistical analysis

#### Prioritization and selection of affected genes

In order to ascertain differentially affected genes between cases and controls we counted the number of cases and controls harboring high impact variants (as defined by VEP). For each gene we counted a) the number of cases with high impact mutations and b) the number of controls with high impact mutations. We then ranked genes according to the greatest difference of counts between cases and controls. To control for differences in batch effects we only considered genes with high impact mutations in both cases and controls. We selected for further analysis only those genes differentially affected (cases vs controls) with a threshold P-value (chi-square) with Bonferroni correction < 4.47E-05 (=0.05 / 1,118 degrees of freedom (df); df= total number of genes: 1,119 - 1).

#### Functional enrichment analysis

Differentially affected genes with P-value < 4.47E-05 were fed into the DAVID gene functional classification tool. This tool’s webserver condensed our list of genes into functionally organized clusters of related genes or biology and useful functional (ontology-defined) annotation to facilitate their interpretation.

#### Analysis of TCR gene cluster variants

The strongest functionally enriched cluster of 12 TCR genes was further analyzed. For that purpose, we identified all high impact variants within these genes and calculated their allele frequencies in cases and controls. P-values using chi-square were calculated to identify genome-wide significant allele frequency differences (P-value < 5.0E-08) between cases and controls. Their deleterious impact using CADD, their consequence and allele frequencies in the European population (using NCBI Allele Frequency Aggregator (ALFA)) were also assessed.

#### Estimation of TCR loss of function via compound heterozygosis

The presence of two different mutated alleles at a particular gene locus may cause complete gene loss of function, a mechanism known as compound heterozygosis. To ascertain whether compound heterozygosis could be present in our study population, we counted high impact allele mutants within each TCR gene. TCR genes with more than 3 heterozygous high impact alleles within the same patient were recorded.

#### Comparison with previously identified genetic markers of severity

Genome-wide association lead variants from the GenOMICC (Genetics of Mortality in Critical Care) study^8^ were retrieved. We focused on variants from this study only, as this is the most complete and recent one researching COVID-19 variants affecting patient severity to date. Observed allele risks from lead GenOMICC variants were counted in our case cohort to calculate their frequency. We only counted variants with sufficient coverage in our gVCFs (Q=> 20). Observed frequencies from GenOMICC lead variants in our case cohort were compared to European NCBI ALFA population frequencies for the same allele risks.

### Additional resources

Additional Supplemental Items are available from Mendeley Data at https://doi.org/10.17632/kxrzh8hgvp.1.

## Data Availability

• COVID-19 whole exome sequencing deidentified data are available from the European Genome-Phenome archive with accession number EGAC00001002480. IBS data from the 1000 Genomes Project is available under the data download portal of the consortium. • The source code developed for the project and associated intermediary data (e.g., phenotype data, VEP outputs) are publicly available in GitHub at https://github.com/manuelcorpas/11-Cov-MadrID. • Any additional information required to reanalyze the data reported in this paper is available from the lead contact upon request.

## Supplemental information

Document S1. Figures S1 and S2 and Table S1

Table S2. Patient clinical histories, related to STAR Methods section ‘Patient Clinical History’

Table S3. Differentially affected genes between cases and controls, related to STAR Methods section ‘Prioritization and Selection of Affected Genes’
