# Supplementary material for: Genetic signature detected in T cell receptors from patients with severe COVID-19
Source: iScience. 2023 Aug 25;26(10):107735. doi: 10.1016/j.isci.2023.107735 (PMC10504482; doi:10.1016/j.isci.2023.107735)
Supplement: Document S1. Figures S1 and S2 and Table S1 [file mmc1.pdf]

iScience, Volume *26*

Supplemental information

Genetic signature detected in T cell receptors from patients with severe COVID-19

Manuel Corpas, Carmen de Mendoza, Víctor Moreno-Torres, Ilduara Pintos, Pedro Seoane, James R. Perkins, Juan A.G. Ranea, Segun Fatumo, Tamas Korcsmaros, José Manuel Martín-Villa, Pablo Barreiro, Octavio Corral, and Vicente Soriano

Supplementary Figures and Legends

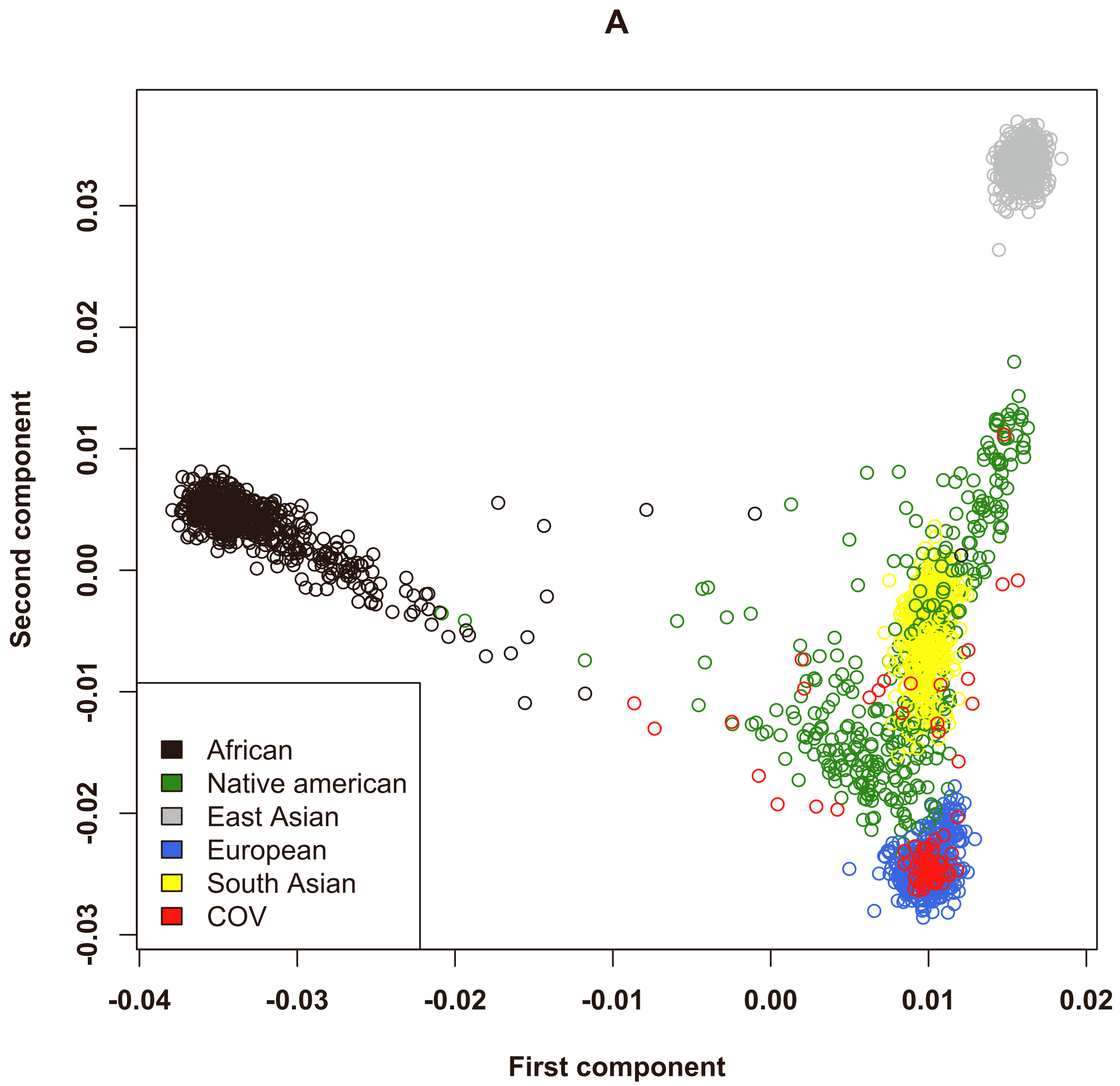

Figure S1**: **Plotting of first and second principal components of 1000G individuals and our 98 COVID-19 cases (red), related to Figure 4. *People from different continents in the 1000G are represented in different colors. The vast majority of cases appear within the 1000G European cluster population (blue).*

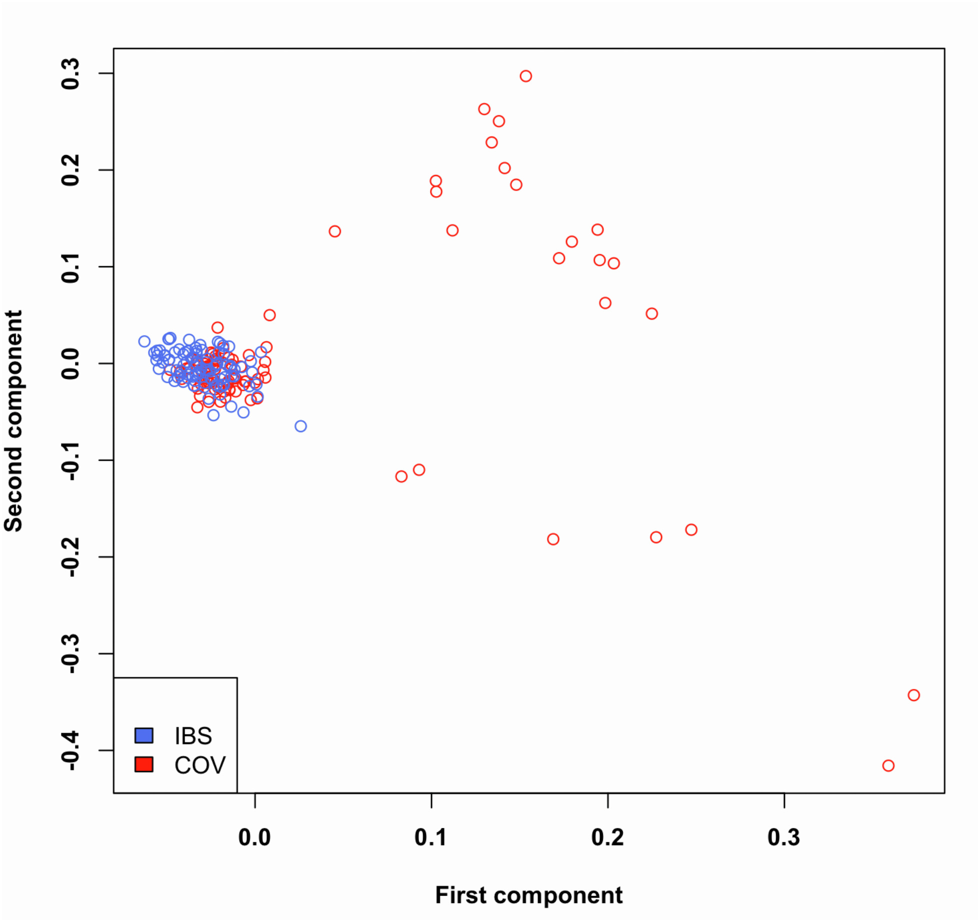

***Figure S2**: **98 cases (red) and 93 controls (blue) principal component analysis, related to Figure 4.** In order to match the genetic ancestries of cases and controls we selected those cases whose principal components clustered within the cluster of controls (left of the perpendicular blue line; N=74).*

Term	Genes	Bonferroni corrected P-value
T cell receptor	TRBV10-1, TRAV19, TRBV7-8, TRBV30, TRBV5-5, TRBV7-3, TRBV7-1, TRBV7-6, TRBV6-7, TRBV7-7, TRBV6-5	5.60E-12
Adaptive immunity	TRBV10-1, TRAV19, TRBV7-8, IGHV3-64, TRBV30, TRBV5-5, TRBV7-3, TRBV7-1, TRBV7-6, TRBV6-7, TRBV7-7, TRBV6-5	4.98E-07
Immunoglobulin domain	TRBV10-1, TRAV19, TRBV7-8, IGHV3-64, TRBV30, TRBV5-5, TRBV7-3, TRBV7-1, TRBV7-6, TRBV6-7, TRBV7-7, TRBV6-5	7.81E-05
Immunity	TRBV10-1, TRAV19, TRBV7-8, IGHV3-64, TRBV30, TRBV5-5, TRBV7-3, TRBV7-1, TRBV7-6, TRBV6-7, TRBV7-7, TRBV6-5	2.07E-04
Receptor	NOTCH2, OR5G3, TRBV10-1, TRAV19, TRBV5-5, TRBV7-3, TRBV7-1, TRBV7-6, TRBV6-7, TRBV7-7, TRBV6-5, TRBV7-8, TRBV30, OR10D3, OR11H7, OR4C5	1.57E-04
Cell membrane	CNTNAP3, NOTCH2, OR5G3, TRBV10-1, TRAV19, IGHV3-64, TRBV5-5, TRBV7-3, TRBV7-1, TRBV7-6, TRBV6-7, TRBV7-7, SLC9B1, TRBV6-5, CLDN5, TRBV7-8, TRBV30, OR10D3, MAL2, OR11H7, OR4C5	0.00126617
Disulphide bond	CNTNAP3, NOTCH2, OR5G3, TRBV10-1, TRAV19, IGHV3-64, TRBV5-5, TRBV7-6, MUC5B, TRBV6-7, TRBV7-7, TRBV6-5, TRBV7-8, CNTNAP3B, TRBV30, OR10D3, OR11H7, OR4C5, GALNT9	0.00283078

Table S1: Functionally enriched terms using DAVID, related to Figure 2*. for significantly different case/control genes, prioritized according to the strength of their statistical term enrichment significance.*
